# Medical and Surgical Management of Postpartum Hemorrhage in a Woman with Factor XIII Deficiency

**DOI:** 10.1155/2016/7963874

**Published:** 2016-08-18

**Authors:** Michael Cheng, Janelle Nassim, Ario Angha, Krisna Srey, Alexander Canales, Chauniqua Kiffin, Yessin Ashmawy, Andrew A. Rosenthal

**Affiliations:** ^1^Florida Atlantic University Charles E. Schmidt College of Medicine, 777 Glades Road, Boca Raton, FL 33431, USA; ^2^Saba University School of Medicine, 27 Jackson Road, Devens, MA 01434, USA; ^3^Division of Acute Care Surgery and Trauma, Memorial Regional Hospital, 3501 Johnson Street, Hollywood, FL 33021, USA; ^4^Division of Women's Health Services, Memorial Regional Hospital, 3501 Johnson Street, Hollywood, FL 33021, USA

## Abstract

Factor XIII deficiency is a rare inherited coagulopathy. Factor XIII is the last clotting factor in the coagulation cascade to insure strength and stability to fibrin clots. Without this enzyme, the fibrous clot is unstable and nonresistant to fibrinolysis. Gravid women with this congenital disease are especially at risk for complications including miscarriages and hemorrhage without appropriate interventions. We present a case of a woman in her 20s with Factor XIII deficiency who was treated with cryoprecipitate and had a successful normal spontaneous vaginal delivery; subsequently, patient suffered from postpartum hemorrhage and consumptive coagulopathy due to consumption of Factor XIII, requiring emergency surgical intervention. Intraoperative management was challenged by an ethical dilemma involving the patient's religious beliefs about not receiving blood. This paper will discuss the mechanism of Factor XIII and the medical and surgical management involved with this patient.

## 1. Introduction

Factor XIII (FXIII) is a transglutimase that circulates in plasma and is activated by thrombin [1]. It plays a key role in the final process of hemostasis in the coagulation pathway by catalyzing the cross-linking of fibrin [1]. This process ensures the strength and stabilization of the clot [2]. The clot also becomes resistant to fibrinolysis with higher concentrations of active FXIII [3]. Inherited FXIII deficiency has an autosomal recessive pattern of inheritance affecting males and females equally, with an estimated incidence of 1 : 3,000,000–1 : 5,000,000 [2]. Prevalence is highest in regions where consanguineous marriage is common, including southeastern Iran [4]. Acquired FXIII deficiency is more common and is caused by an autoantibody that binds to FXIII interfering with its main function [2]. Without cryoprecipitate and factor infusion interventions, FXIII deficiency results in delayed wound healing and recurrent spontaneous miscarriages [2, 5]. In addition to this rare coagulopathy, this patient's case was complicated by her religious beliefs about accepting blood products as a Jehovah's Witness.

## 2. Case Presentation

A South American Jehovah's Witness in her mid-20s with significant past history of two first-trimester spontaneous abortions, hypothyroidism, unilateral renal agenesis, and FXIII deficiency presented to our hospital at 41 weeks' gestation, with perineal pain and contractions. She started her prenatal care and received prophylactic cryoprecipitate every two weeks to treat her FXIII deficiency abroad, prior to arriving in the United States at 37 weeks' gestation. Upon admission to the Labor and Delivery Unit, consultation with Maternal Fetal Medicine and Hematology recommended proceeding with a vaginal delivery. She received eight units of prophylactic cryoprecipitate; patient had a successful normal spontaneous vaginal delivery of a live infant with Apgar of nine at one minute and nine at five minutes. Following delivery, abrupt hemorrhage occurred soon after evacuation of the placenta. The hemorrhage was thought to be due to retained placental elements or uterine trauma, because the uterus was appropriately contracted following initial delivery. In this immediate postdelivery period of 20–30 minutes, the patient's blood loss was underappreciated and was subsequently estimated at 2 L. After lengthy discussion with the patient and her family, patient consented to receive blood products in the event of life-saving necessity. A Massive Transfusion Protocol was initiated; the patient received approximately 2.5 L of crystalloid while blood products were prepared. She was transfused eight additional units of cryoprecipitate, four units of fresh frozen plasma, three units of packed red blood cells, and one unit of platelets. A continuous infusion of aminocaproic acid was chosen over intermittent dosing of tranexamic acid (TXA) to provide a steady more consistent presence of antifibrinolytic activity; this continued until FXIII was available several days later.

The patient remained hypotensive and was taken to the operating room for hemorrhage control; an emergency supracervical hysterectomy was performed. Continued bleeding was noted from the visceral surfaces and Trauma Services was consulted intraoperatively for damage control. The pelvis was packed, drains were placed, and a temporary closure was performed. The plan was to stabilize the patient and reverse her coagulopathy. The packing was removed and delayed closure was performed on postoperative day two. Patient was subsequently discharged on postoperative day number six.

## 3. Discussion

While, in some rare instances, women with FXIII deficiency are able to successfully deliver live newborns without FXIII replacement therapy [6], FXIII deficiency is associated with pregnancy complications including miscarriage, antepartum hemorrhage, and postpartum hemorrhage [6]. One of these complications is postpartum hemorrhage, defined as blood loss exceeding 500 mL in the first 24 hours following vaginal delivery or blood loss exceeding 1000 mL in the first 24 hours following caesarean delivery [7]. Postpartum hemorrhage is the leading cause of maternal mortality [8], responsible for an estimated 25% of maternal deaths associated with childbirth [9]. Management of the gravid patient with FXIII deficiency is focused on prophylaxis with FXIII concentrate, cryoprecipitate, or fresh frozen plasma, with FXIII concentrate being preferred when available [6, 10]. The exact concentration of FXIII in the blood necessary for optimal pregnancy outcomes is unknown; however, a systematic review of pregnancy outcomes in women with FXIII deficiency found that, in pregnancies reaching viability, a median FXIII concentration of 12 IU/dL in the blood was maintained during pregnancy and a median FXIII concentration of 35 IU/dL was maintained during labor and delivery [6]. While there is no universal consensus about the optimal dosing regimen for FXIII concentrate in pregnancy, administering 250 IU weekly up until the 23rd week of pregnancy, followed by 500 IU weekly up until labor and delivery, with a 1000 IU booster dose has been found to be effective [6]. Due to its approximately two-week half-life, those receiving prophylactic therapy with FXIII concentrate should have protective coverage extending into the postpartum period [6, 10]. In this case, due to the inability to obtain FXIII prior to delivery, cryoprecipitate was used instead. Additional crystalloid infusion was recognized as a potential contributor to dilutional coagulopathy, but, at the time, hypotension and the required induction of anesthesia required aggressive volume resuscitation.

Our patient's condition was further complicated when she experienced disseminated intravascular coagulation (DIC). DIC can be categorized into either overt or nonovert based on the criteria set out by the International Society of Thrombosis and Hemostasis (ISTH) [11, 12]. The scoring takes into account platelet count, prothrombin time, the amounts of fibrinogen, and D-dimer (Table 1) [12]. Overt DIC, or the stage at which a patient becomes decompensated, is considered with a score greater than or equal to five [12]. A study conducted by Song et al. [13] set out to investigate the correlation between FXIII and DIC. Their study population consisted of patients with DIC from conditions ranging from malignancies to infections. After measuring FXIII levels, the team discovered that plasma levels were significantly decreased in overt DIC patients compared to nonovert DIC patients. DIC scores were inversely correlated to FXIII activity [13]. It can therefore be deduced that those with lower FXIII activity, such as our patient, may be more likely to reach the decompensating stage of DIC and thereby suffer more severe hemorrhage.

When medical management fails to reverse coagulopathy, surgical management must be considered as a life-saving procedure. Intraoperatively, the patient had lost 3 L of blood, an intraoperative consult to Trauma Services was obtained to perform damage control surgery (DCS). DCS is a surgical strategy frequently used by trauma surgeons to control surgical bleeding in patients with multiple visceral and/or vascular injuries. Damage control is often life-saving in critical surgical patients; a review by Shapiro et al. concluded that up to 60% of patients survive with this approach [14]. DCS is practiced in a three-stage approach [14]. The first stage is the initial laparotomy that aims to quickly stop hemorrhage by packing, suturing, ligation, or balloon tamponade [14, 15]. Balloon tamponade and B-Lynch suture compression were considered as temporizing maneuvers; ultimately, however, expeditious exploration and hysterectomy were chosen over potentially time-consuming attempts at uterine salvage. The main goals of DCS are to avoid a downward spiral of the “lethal triad” (hypothermia, metabolic acidosis, and refractory coagulopathy) and to limit operative time so the patient can be transferred to the surgical Intensive Care Unit (ICU), which is stage II of DCS, for physiologic stabilization [14, 16, 17]. Once the patient is stabilized in the ICU, including optimization of hemodynamic status, reversal of coagulopathy, and fluid resuscitation, the patient is brought back to the operating room for stage III of the DCS strategy, which includes removal of the packing, definite repair of injuries, and definitive abdominal closure [14, 17]. In our case, the major element of the damage control surgery included pelvic packing to stop the massive hemorrhage.

A variety of techniques exist for pelvic packing, with the most common one being insertion of absorptive materials, such as gauze rolls, into the abdomen and pelvis for tamponade of the bleeding and to provide sufficient pressure to achieve hemostasis. However, there is a delicate balance between packing the abdomen too tight, which may result in compartment syndrome, and not packing tight enough, which can lead to continued hemorrhage after packing is placed. Complications from overly forceful packing can include neuropathies, compression of the inferior vena cava (IVC), and renal failure secondary to IVC compression. As a result of an intentionally retained foreign body, febrile morbidity and sepsis are also concerns [18].

Jehovah's Witnesses decision to abstain from blood or blood fractions is a personal choice based on individual religious beliefs. Administration of blood products against the wishes of a patient is a potentially criminal act that could be subject to prosecution [19]. These forbidden blood products include red cells, white cells, platelets, and plasma. Blood fractions, including cryoprecipitate and individual clotting factors, are acceptable to some Jehovah's Witnesses, including the patient in this case [19]. In the past, our patient had previously accepted prenatal infusion of cryoprecipitate. In the postpartum state, she initially refused transfusion of other blood products but eventually consented to receive red blood cells, plasma, and platelets due to the severity of her condition with postpartum hemorrhage and DIC requiring an emergent supracervical hysterectomy and DCS. She also consented to receive recombinant FXIII in the postpartum state. Ideally, a patient's final decision about blood products would be made prior to any complications so the treatment team can plan accordingly; however, we recognize that emergent situations similar to this case may occur.

This case also highlights the unique patient population treated at our hospital in South Florida, in which a large percentage of our patients are international patients from either Latin America or the Caribbean. While several international and national registries have been established for rare bleeding disorders [20, 21] the true incidence of rare bleeding disorders such as Factor XIII in Latin American populations may be underestimated due to the fact that international registries draw heavily from data gathered from Europe, North America, and Iran [21].

## Figures and Tables

**Table 1:** International Society of Thrombosis and Hemostasis (ISTH) criteria for overt versus nonovert DIC. A score greater than or equal to 5 is compatible with overt DIC whereas a score less than 5 is suggestive for nonovert DIC.

Platelet count (/*μ*L)	<100000	1 point
	<50000	2 points

Prolongation of PT (s)	>3 but <6	1 point
	>6	2 points

Fibrinogen (mg/dL)	100	1 point

D-dimer (*μ*g/mL)	0.5–1	1 point
	1–3	2 points
	≥3	3 points
